# The Convoy Corps in the Balkans

**Published:** 1914-02-07

**Authors:** 


### WOMEN AND WAR.

### The Convoy Corps in the Balkans.

"Four things greater than all things are" declared Rudyard Kipling. First on the list he put women, and last, war; but no indication was given whether the arrangement was in ascending or descending order. "Militancy" has already shown us that war and women are not so far divorced as our forefathers thought them; but the author of a recently published book * carries the argument a stage further by pleading for the formation of women's units in the Territorial Force. Mrs. Stobart, after Colonial experiences which familiarised her with outdoor life and imbued her with a contempt for conventionality, organised some four or five years ago, in London, a small force of women known as the Women's Convoy Corps. This Corps was annually trained in camp on lines essentially similar to those of the Territorial Field Ambulances—that is to say, the members lived under canvas, cooked their own food, drove their own wagons, and, in fact, "roughed it" like men. The training of the Corps was especially directed to the filling of the gap in the Territorial Force organisation which exists between the field ambulances and the base hospitals, a gap which officially is supposed to be filled by units of the British Red Cross Society known as Voluntary Aid Detachments. These latter bodies consist of well-intentioned but most imperfectly instructed men and women who would, in Mrs. Stobart's judgment (and there is much to be said in favour of her view), be more of a hindrance than a help in war-time. She inaugurated the Convoy Corps to show that women can devise a more excellent system; and the War Office was a consenting party to the experiment.

### No Women Need Apply.

By the time Mrs. Stobart had got her Corps into thorough working order, the Balkan War broke out—more than a year ago; and she at once sent in an application to the Red Cross Society to be accepted, with some of the women in her Corps, as volunteers. She was refused on the ground that the Society was sending none but men to attend the sick and wounded. It should be mentioned that Mrs. Stobart and her friends were all registered with the Red Cross Society, whereas many of the men whom that Society sent were unregistered and innocent of even such training as a Volunteer Aid Detachment can give. So Mrs. Stobart went to Bulgaria herself, saw the authorities there, and telegraphed home for a specially selected detachment to join her. Accordingly, sixteen women went to the front, including three women doctors; and at Kirk Kilisse they conducted a stationary hospital most successfully for seven weeks.

### Women and the Territorial Army.

The record and the experiences of the detachment are now told by the leader, who propounds some novel ideas on the strength of her own and her comrades' accomplishments. She suggests that the Royal Army Medical Corps of the Territorial Force should enlist women and organise them in properly trained units. There is little, if any, of the work now performed by men of the R.A.M.C. (she says) which could not be done by women; and, while there is a shortage in Territorial numbers, it is wasteful to draw off able-bodied men from the fighting line to do any work which could be done by women. If woman is trained and disciplined, as Mrs. Stobart would like, so as to be of practical service to the country, she must be rewarded by pay, rank, and in fact by due recognition such as men receive. That is what Mrs. Stobart asks, and what her whole book is intended to promote.

It need hardly be said that such a large demand needs careful deliberation. It is easy to formulate a few minor objections. Thus the work which Mrs. Stobart did was not Convoy Corps' work at all, but that of a stationary hospital: on the one trek which she had to undertake, some forty (male) drivers had to be found to transport sixteen ladies and their stores. No one doubts that women doctors and women nurses can staff a hospital. Women cooks were more of a novelty, but even Mrs. Stobart's hospital required male assistance for such necessary services as removal of refuse, latrine digging, and so forth. Again, because Mrs. Stobart has very remarkable force of character, and her small band did gallant deeds, it does not follow that large numbers of women of equal capacity exist in Britain who have sufficient leisure, means, and inclination to carry them through four years of training in the Women's Convoy Corps. Mrs. Stobart holds that such women abound; it is quite possible, but it is not proved.

These, as has been noted, are minor matters. The principle Mrs. Stobart contends for is the duty of women to take part in national defence. Here, in our opinion, she is beating the air to some extent. For there is no official recognition in this country of the duty of anyone, male or female, to help to defend the country. Moreover, trained women are admitted to the Army, though it is the Regulars, not the Territorials, who enjoy the ministrations of Queen Alexandra's Imperial Military Nursing Service. When Mrs. Stobart denounces the imperfect training and general inefficiency of many of the Voluntary Aid Detachment units, for which the British Red Cross Society is responsible, she is doing good service: the reason for it is mainly the fault of Lord Haldane's Territorial scheme, which left the provision of this essential part of R.A.M.C. work to

* *War and Women*. By Mrs. St. Clair Stobart. G. Bell and Sons. 1913. Pp. 239. Price 3s. 6d. net.

voluntary, unsubsidised, and ineffective agencies. When she is urging the duty of national defence, again she carries our sympathies. But not infrequently she relapses into mere childishness, and her habit of italicising about 2 per cent. of her words does not give point to her arguments. This is all the greater pity in that her criticisms of the British Red Cross Society for refusing to send women nurses are probably justified, but will fail of their due effect owing to the irrationality of which this book affords so many glaring examples.

Let us take one instance in point of this. The Bulgarians, as everyone knows, drove the Turks headlong through Thrace to Chataldja, and surprised the world by their rapid and overwhelming victories. "No better argument," says Mrs. Stobart (p. 170), "against a professional as compared with a 'territorial' army could well . . . have been afforded than by this war in which by far the larger amount of professionalism was on the losing side." The inverted commas round the word territorial are hers, not ours; and if words mean anything at all, this passage means that Mrs. Stobart thinks our civilian soldiers can hold their own against the Regular Army. Such a view makes one disinclined to trust Mrs. Stobart's judgment; but the implication in the passage and its context that the Bulgarians won because the Turks had lost faith in their religion, and not because they themselves were better trained, is so contrary to all the known circumstances that it makes one want to throw the book into the waste-paper basket straightaway. One resists the temptation only because her criticisms of the voluntary aid detachments are of real value, and because she presents us with novel ideas.

